# Association of gut microbiota and inflammatory markers with enteral nutrition intolerance in patients with early-stage moderate-to-severe intracerebral hemorrhage

**DOI:** 10.1128/spectrum.03138-25

**Published:** 2026-05-29

**Authors:** Haixiao Jiang, Hengzhu Zhang, Xuyuan Ding, Youwe Wang, Chenyi Wu, Aijun Peng, Demao Cao, Jiahao Wang, Wei Zeng, Fei Zhu

**Affiliations:** 1 Department of Neurosurgery, The Affiliated Hospital of Yangzhou University, Yangzhou, China; 2 Department of Neurosurgery, Northern Jiangsu People's Hospital 619558, Yangzhou, China; 3 Department of Neurosurgery, Northern Jiangsu People’s Hospital Affiliated to Yangzhou University, Yangzhou, China; 4 Department of Neurosurgery, Taizhou Second People’s Hospital Affiliated to Yangzhou University, Taizhou, China; 5 Department of Neurosurgery, Yancheng First Hospital, Affiliated Hospital of Nanjing University Medical School 612638, Yancheng, China; 6 Department of Neurosurgery, The First People's Hospital of Yancheng, Yancheng, China; Tainan Hospital Ministry of Health and Welfare, Tainan, Taiwan (Province of China)

**Keywords:** enteral nutrition intolerance, gut microbiome, biomarker, inflammatory markers, intracerebral hemorrhage

## Abstract

Enteral nutrition intolerance (ENI) is a common and challenging complication in patients with moderate-to-severe intracerebral hemorrhage (msICH). Emerging evidence suggests that gut microbiota is vital for host immune regulation and nutrient metabolism. However, the relationship between intestinal microbial alterations, inflammatory responses, and ENI in patients with early-stage msICH (emsICH) remains poorly understood. Therefore, this study aimed to explore these associations. A total of 100 patients with emsICH were enrolled in this study, including 50 with ENI and 50 without ENI (NENI). The gut microbiota composition was assessed using 16S ribosomal RNA gene sequencing, and the relationships between microbial profiles, systemic inflammatory indicators, and ENI status were systematically analyzed. Patients with ENI exhibited significantly reduced alpha diversity and distinct microbial structure when compared to those with NENI. Linear discriminant analysis effect size identified 19 differentially abundant taxa, with pathogenic genera, including *Enterococcus* and *Finegoldia*, enriched in patients with ENI. However, beneficial short-chain fatty acid-producing genera, such as *Subdoligranulum* and *Faecalibacterium*, were enriched in patients with NENI. A six-genus biomarker panel demonstrated a strong predictive power for ENI (area under the curve = 0.812). Functional phenotype analysis revealed an increased abundance of Gram-negative and facultative anaerobic bacteria in patients with ENI. Moreover, ENI-associated genera were positively correlated with blood inflammatory indicators, whereas NENI-associated taxa exhibited negative correlations. The analysis of the Kyoto Encyclopedia of Genes and Genomes pathways suggested that several essential physiological pathways in ENI were disturbed, with upregulation of inflammation-related signaling pathways (e.g., Jak-STAT and MAPK) and downregulation of pathways involved in metabolism (particularly in energy metabolism). Gut microbiota dysbiosis in patients with emsICH is strongly associated with ENI and systemic inflammation. Specific microbial signatures may serve as non-invasive biomarkers for ENI risk assessment. These findings shed light on gut microbiota-associated inflammation and the ENI axis and offer a theoretical basis for microbiota-targeted and nutritional interventions in neurocritical care.

**IMPORTANCE:**

Enteral nutrition intolerance (ENI) is a frequent and serious complication in patients with moderate-to-severe intracerebral hemorrhage (ICH). Our study demonstrates that ENI is strongly associated with gut microbiota imbalance, characterized by an overrepresentation of inflammation-promoting bacteria and a loss of beneficial short-chain fatty acid-producing taxa. This microbial shift was closely linked to heightened systemic inflammation, providing new insights into the gut-inflammation-ENI axis in neurocritical illness. By identifying specific bacterial signatures, our findings suggest that stool-based microbial assessment may serve as a noninvasive tool for early risk prediction and highlight the potential of microbiota-targeted strategies to improve nutritional management and outcomes in patients with ICH.

## INTRODUCTION

Intracerebral hemorrhage (ICH) is among the most dangerous acute cerebrovascular illnesses in neurosurgery, as it poses a serious health risk (1). Its features include a sudden onset, swift progression, and high rates of mortality and disability (1). Patients with moderate to severe grades of ICH are exceptionally at malnutrition risk due to altered consciousness levels, impaired swallowing, and excessive physiological stress (1, 2). A timely start of enteral nutrition support is required to prevent poor overall clinical outcomes caused by the breakdown of the intestinal wall, poor nutritional status, and weakened immune system. Almost 30%–50% of patients with ICH have enteral nutrition intolerance (ENI), which is mostly described by gastric retention, abdominal distension, or diarrhea (3–5). ENI could increase the risk of infection and complications, prolong recovery, and lack early specific symptoms. Hence, studying the underlying mechanisms of ENI and devising accurate predictive models would allow for better stratification and the development of personalized nutritional approaches.

It appears that patients in neurocritical care frequently exhibit systemic inflammatory response syndrome or some form of infection (6, 7). This is often accompanied by elevated levels of pro-inflammatory C-reactive protein (CRP), procalcitonin (PCT), and interleukin-6 (IL-6) (6). These inflammatory mediators could affect gastrointestinal motility and secretion through activation of the enteric nervous system and suppression of hormone secretion, leading to an increased risk of nutrient intolerance (8, 9). In parallel, there is greater support for a robust systemic inflammation and gut microbiota relationship. The human gut microbiota is a dynamic ecosystem comprising trillions of microorganisms. Beyond nutrient metabolism and digestion, these microorganisms significantly influence the development and function of the host immune system (10, 11), thereby exerting a substantial impact on systemic inflammatory responses (12). Multiple studies have suggested that gut microbiota dysbiosis may impair intestinal barrier function and cause gastrointestinal motility disorders (13–15), which could contribute to the development of ENI. As a result, modulating the gut microbiota is receiving more attention as a possible therapeutic approach to manage ENI.

For instance, Wang Y et al. (16) demonstrated that ENI could be prevented alongside a reduction in mortality rates through prebiotic and probiotic supplementation. While there is a significant amount of literature related to risk factors and predictors of ENI, patients with neurological critical illnesses are underrepresented. There is a dearth of research focusing on early-stage moderate-to-severe intracerebral hemorrhage (emsICH). Exploring the interaction of the gut microbiota and emsICH with ENI could elucidate potential novel strategies for avoiding ENI. This is the gap that the current study aimed to fill.

This study aimed to assess patients with emsICH who present with ENI by characterizing their gut microbiota and inflammatory response profiles. We intended to study the complicated relationships among them and develop a predictive model for ENI using microbial biomarkers. Through the creation of a quantifiable predictive tool, we aimed to enhance early risk stratification and targeted interventions. This study also aimed to lay the groundwork for therapies focused on the microbiota, strategic nutritional interventions, and, eventually, new treatment paradigms for patients suffering from emsICH.

## MATERIALS AND METHODS

### Study design and population

**Fig 1:**
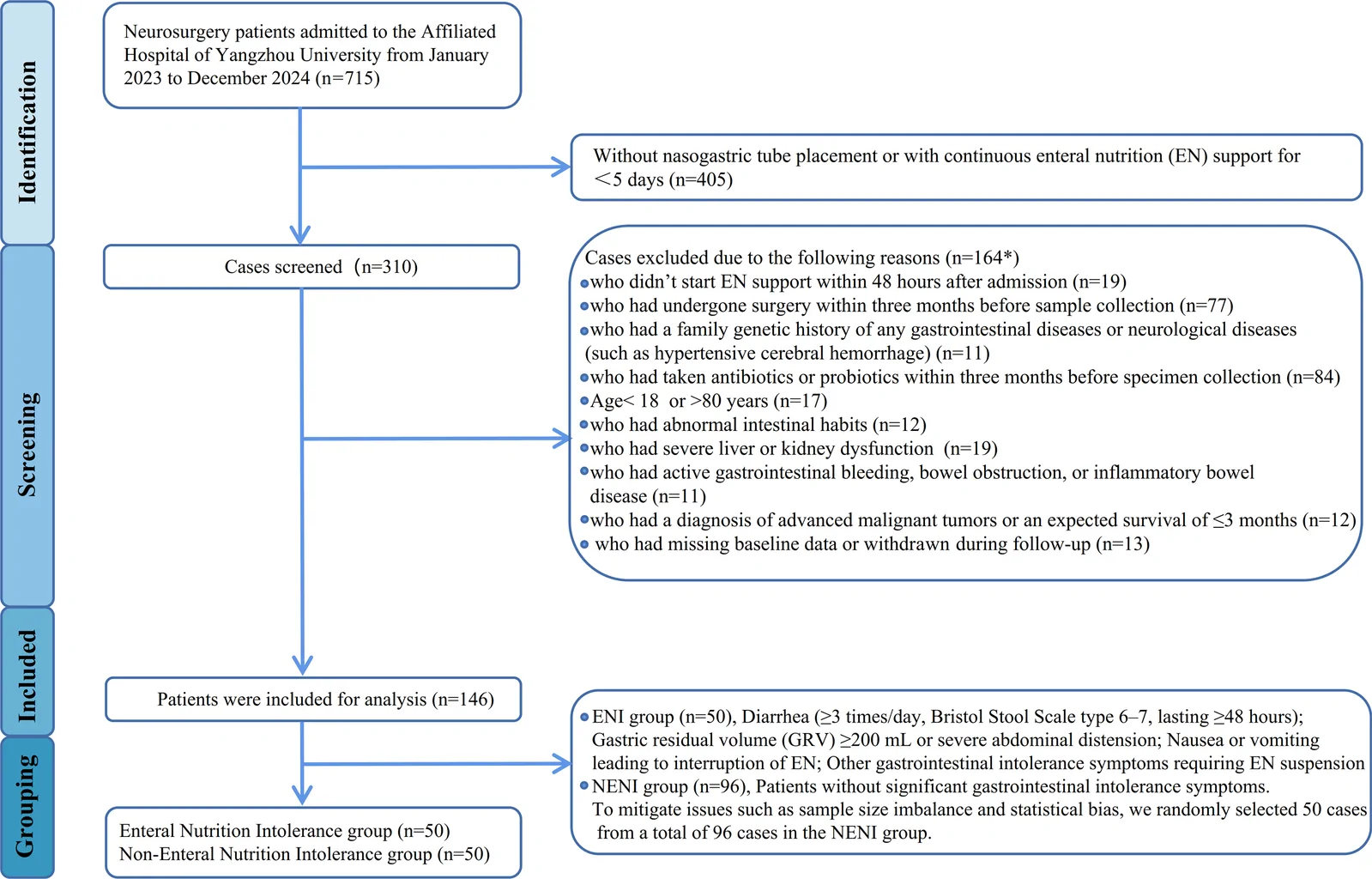
Flowchart of patient screening and selection.

This study included patients with emsICH. All patients were admitted to the Affiliated Hospital of Yangzhou University between January 2023 and April 2025. All participants were diagnosed with ICH within 24 h of symptom onset, and all diagnoses were validated using X-ray computed tomography. The inclusion and exclusion criteria for patient screening are presented in Fig. 1. The written informed consent was obtained for 100 patients, including 50 with ENI and 50 without ENI (NENI). Clinical information, including demographic information and serum inflammatory indicators, was obtained for all subjects. The inflammatory indicators obtained were neutrophil-to-lymphocyte ratio (NLR), monocyte-to-lymphocyte ratio (MLR), serum amyloid A (SAA), IL-6, PCT, and high-sensitivity C-reactive protein (hs-CRP).

### Sample collection and storage

Sterile swabs were used to collect fresh fecal samples from all subjects within 12 h of hospital admission. The samples were briefly dissolved in the primary preservation solution and immediately frozen at −80°C until DNA extraction. DNA was extracted using a PowerMax DNA Isolation Kit following the manufacturer’s protocol. A NanoDrop ND-1000 spectrophotometer (Thermo Fisher Scientific) was used to determine DNA concentration and quality. Blood samples were also collected immediately for routine tests and assessment of inflammatory indicators after collecting stool specimens.

### 16S ribosomal RNA (rRNA) Gene Sequencing

The V4 region of the bacterial 16S rRNA gene was amplified by polymerase chain reaction (PCR) using specific primers: 515F (5′-GTGCCAGCMGCCGCGGTAA-3′) and 806R (5′-GGACTACHVGGGTWTCTAAT-3′). Each sample had a unique 7 bp barcode embedded in the sequence that allowed the samples to be distinguished during multiplex sequencing analyses. PCR amplification was performed under strictly defined conditions, including 30 s at 98°C for the initial denaturation, 25 cycles of denaturation at 98°C (15 s), annealing at 58°C (15 s), and extension at 72°C (15 s), followed by a final extension at 72°C for 1 min. After completion of PCR amplification, the products were purified with AMPure XP Beads (Omega Bio-Tek, USA) to remove any impurities. The purification of the PCR products was quantified using the PicoGreen dsDNA Assay Kit (Invitrogen, USA) to analyze the DNA concentration. Once quantification was complete, equal volumes of purified amplicons from each sample were combined into a pooled sample, which was then sequenced using the Illumina HiSeq 4000 platform, set to generate 2 × 150 bp paired-end reads, providing a comprehensive analysis of the amplified 16S rRNA gene regions.

### Microbiota data analysis

The raw sequencing reads were demultiplexed and sorted into data sets for individual samples using barcodes and primer sequences. After sorting, the barcodes and primer sequences were removed. Paired-end reads were then merged for each sample using the VSEARCH software (version 2.15.0 [17]), resulting in raw tags. Quality control was conducted to filter out low-quality reads using the QIIME2 software (version 2022.2) (18, 19), as low-quality reads could impact the results. After quality control, the sequences were clustered into amplicon sequence variants (ASVs) using CD-HIT software (version 4.6.1) with a 100% sequence similarity threshold (20). To remove possible biases associated with differences in sequencing depth across samples, ASV counts were normalized. All samples were retained and adjusted to have the same number of total ASV sequences and then converted to relative abundances (summing to 1). A representative sequence was selected to represent each ASV based on the default parameters. The representative sequences and the ASV table were imported into QIIME2, where all ASVs with relative abundances below 0.001% across all samples were removed. Taxonomic classification of ASVs was performed using the pre-trained classifier in QIIME2. The taxonomy was collapsed from the broad phylum level down to the genus level using the "qiime taxa collapse" command to ultimately create a more informative ASV list. Microbial diversity and richness of each sample were assessed using several alpha diversity indices, including Shannon, Simpson, Chao1, Observed Species, ACE, PD whole tree, and Goods coverage from the ASV table generated in QIIME2 (21). Beta diversity analysis was performed using the Bray–Curtis dissimilarity metric to determine the differences between microbial communities across groups. Differences between samples were visualized using principal coordinate analysis (PCoA) and non-metric multidimensional scaling (NMDS) (22). Differences between groups were assessed for significance using analysis of similarities (ANOSIM), which enabled a robust interpretation of the statistical results. To determine the taxa associated with ENI, we performed linear discriminant analysis effect size (LEfSe [23]) with linear discriminant analysis (LDA) scores greater than 3.0 (24). Microbial functional profiles were inferred using the Phylogenetic Investigation of Communities by Reconstruction of Unobserved States (PICRUSt [25]) method based on the Kyoto Encyclopedia of Genes and Genomes (KEGG) database (26, 27). We further established the microbial community phenotypes using the BugBase tool (28).

### Statistical analysis

Statistical analyses were conducted using R software (version 4.1.0) and the Statistical Package for the Social Sciences software (version 26.0). Continuous data is expressed as mean ± standard deviation. Between-group differences for continuous data were determined using an independent samples *t*-test for normally distributed data or the Mann–Whitney *U* test for non-normally distributed data. Categorical data is presented as frequencies and percentages (%), and between-group comparisons were performed using the chi-square test or Fisher’s exact test, as appropriate. Spearman’s rank correlation analysis was used to determine the association between changes in gut microbiota and serum inflammatory indicators. The predictive ability of potential biomarkers was assessed using receiver operating characteristic (ROC) curve analysis.

## RESULTS

### Summary of participant clinical parameters

**Fig 2:**
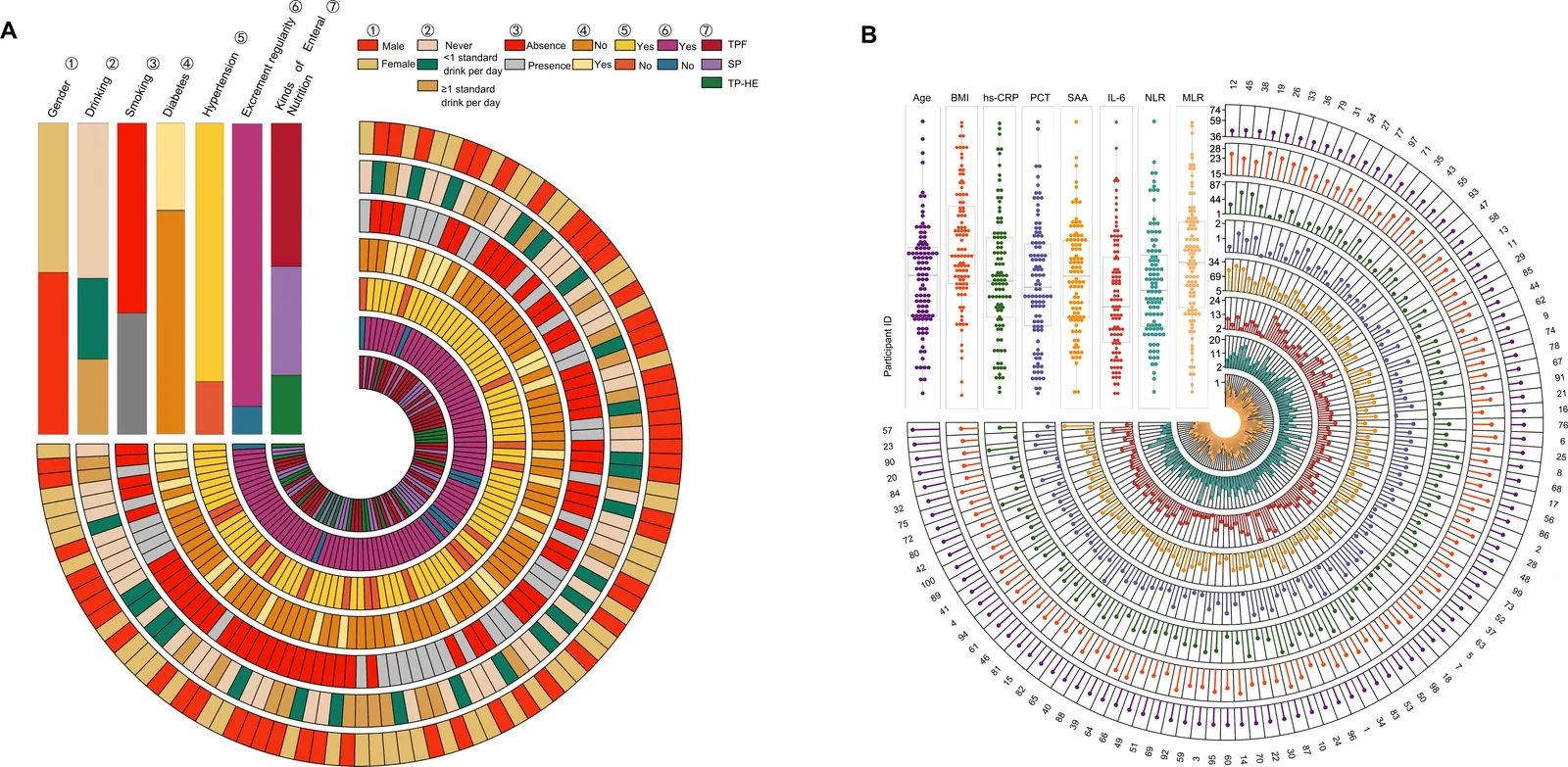
Demographic characteristics (**A**) and clinical measurements (**B**) of 100 patients.

Table 1 summarizes the clinical characteristics of the enrolled participants. Figure 2 displays more detailed information regarding the demographics of all participants. Importantly, the participants in the two groups were demographically equivalent, demonstrating that the groups did not diverge, allowing a balanced baseline at the start of the study.

**TABLE 1:** The baseline characteristics of all participants^*a*^

	ENI	NENI	ENI vs NENI
	(*n* = 50)	(*n* = 50)	*P-value*
Age (Mean ± SD)	55.90 ± 6.02	57.98 ± 5.42	0.0730
Gender			0.423
Male	28 (56%)	24 (48%)	
Female	22 (44%)	26 (52%)	
BMI	23.68 ± 2.05	22.98 ± 2.30	0.112
Smoking			0.539
Absence	32 (64%)	29 (58%)	
Presence	18 (36%)	21 (42%)	
Drinking			0.373
Never	23 (46%)	27 (54%)	
<1 standard drink per day	15 (30%)	14 (28%)	
≥1 standard drink per day	12 (24%)	9 (18%)	
Hypertension			0.424
Positive	40 (80%)	43 (86%)	
Negative	10 (20%)	17 (34%)	
Diabetes			0.656
Negative	35 (70%)	37 (74%)	
Positive	15 (30%)	13 (26%)	
Excrement regularity			0.727
Yes	46 (92%)	45 (90%)	
No	4 (8%)	5 (10%)	
Kinds of enteral nutrition			0.919
Enteral nutritional suspension (TPF)	24	22	
Enteral nutritional suspension (SP)	17	18	
Enteral nutritional emulsion (TP-HE)	9	10	
hs-CRP (mg/L, Mean ± SD)	41.24 ± 9.66	35.83 ± 7.55	0.182
PCT (ng/mL, Mean ± SD)	0.91 ± 0.28	0.80 ± 0.21	0.231
SAA (mg/L, Mean ± SD)	62.43 ± 19.09	57.02 ± 17.74	0.281
IL-6 (pg/mL, Mean ± SD)	10.26 ± 3.25	8.67 ± 2.30	0.102
NLR (Mean ± SD)	9.73 ± 2.62	8.41 ± 2.68	0.074
MLR (Mean ± SD)	0.75 ± 0.17	0.69 ± 0.16	0.085

^*a*^ ENI, enteral nutrition intolerance; NENI, non-enteral nutrition intolerance; BMI, body mass index; hs-CRP, high-sensitivity C-reactive protein; PCT, procalcitonin; SAA, serum amyloid A protein; IL-6, interleukin-6; NLR, neutrophil-to-lymphocyte ratio; MLR, monocyte-to-lymphocyte ratio; SD, standard deviation.

### Distribution of microbiota in ENI versus patients with NENI

**Fig 3:**
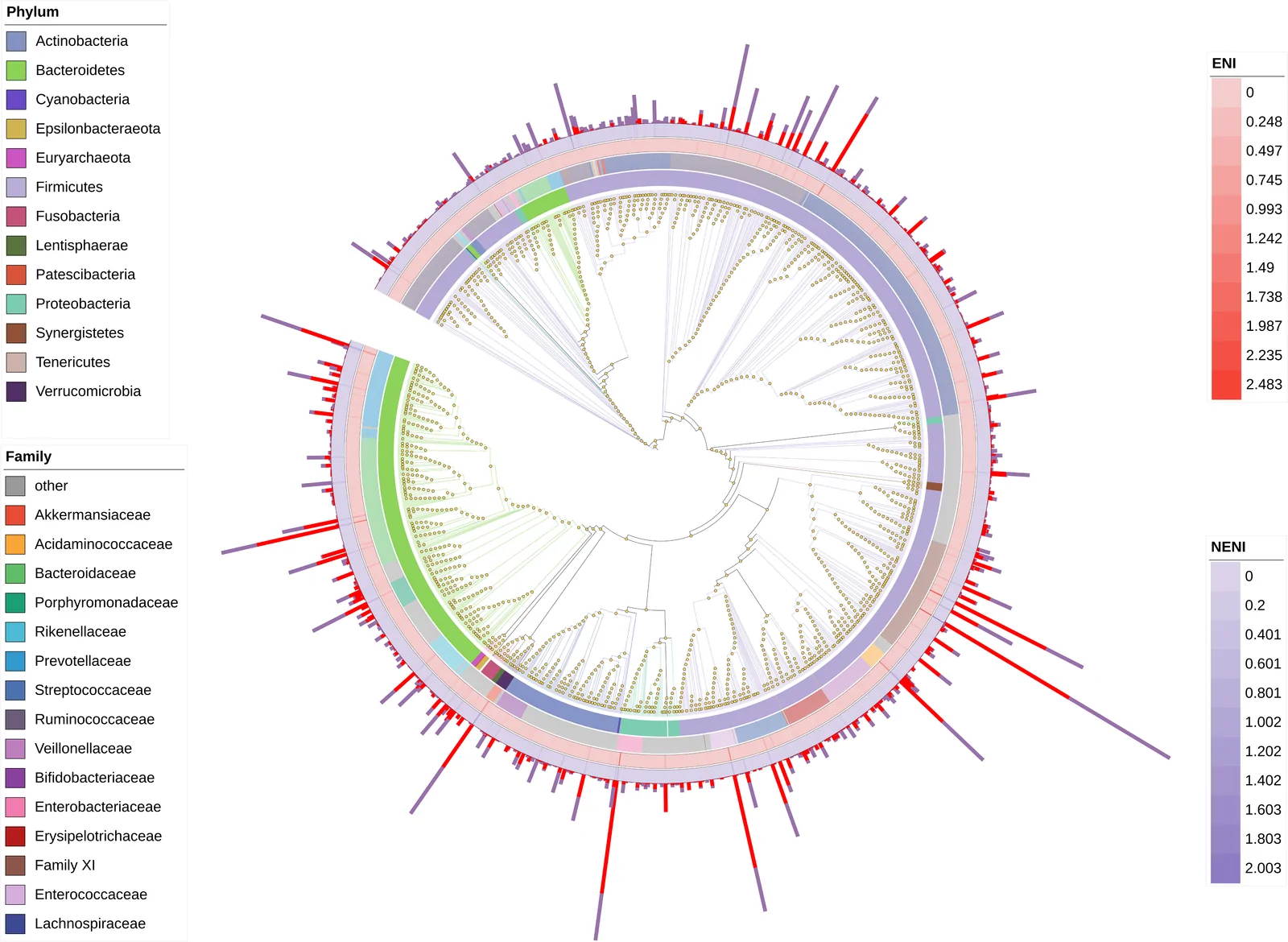
Phylogenetic tree and distribution of gut microbial community. The innermost branches depict the phylogenetic relationships among bacterial taxa derived from 16S rRNA gene sequences. Each tip represents a taxon with clustering based on sequence similarity. From inside to outside, the four concentric rings surrounding the tree are displayed. The innermost two concentric circles represent the phylum and family to which each ASV belongs, while the outermost two concentric circles represent the relative abundance of ASV in ENI (red) and NENI (purple), with darker colors indicating higher concentrations. Additionally, the outermost bar chart provides a more intuitive visualization of the abundance differences between groups. Abbreviation: ASV, amplicon sequence variant.

**Fig 4:**
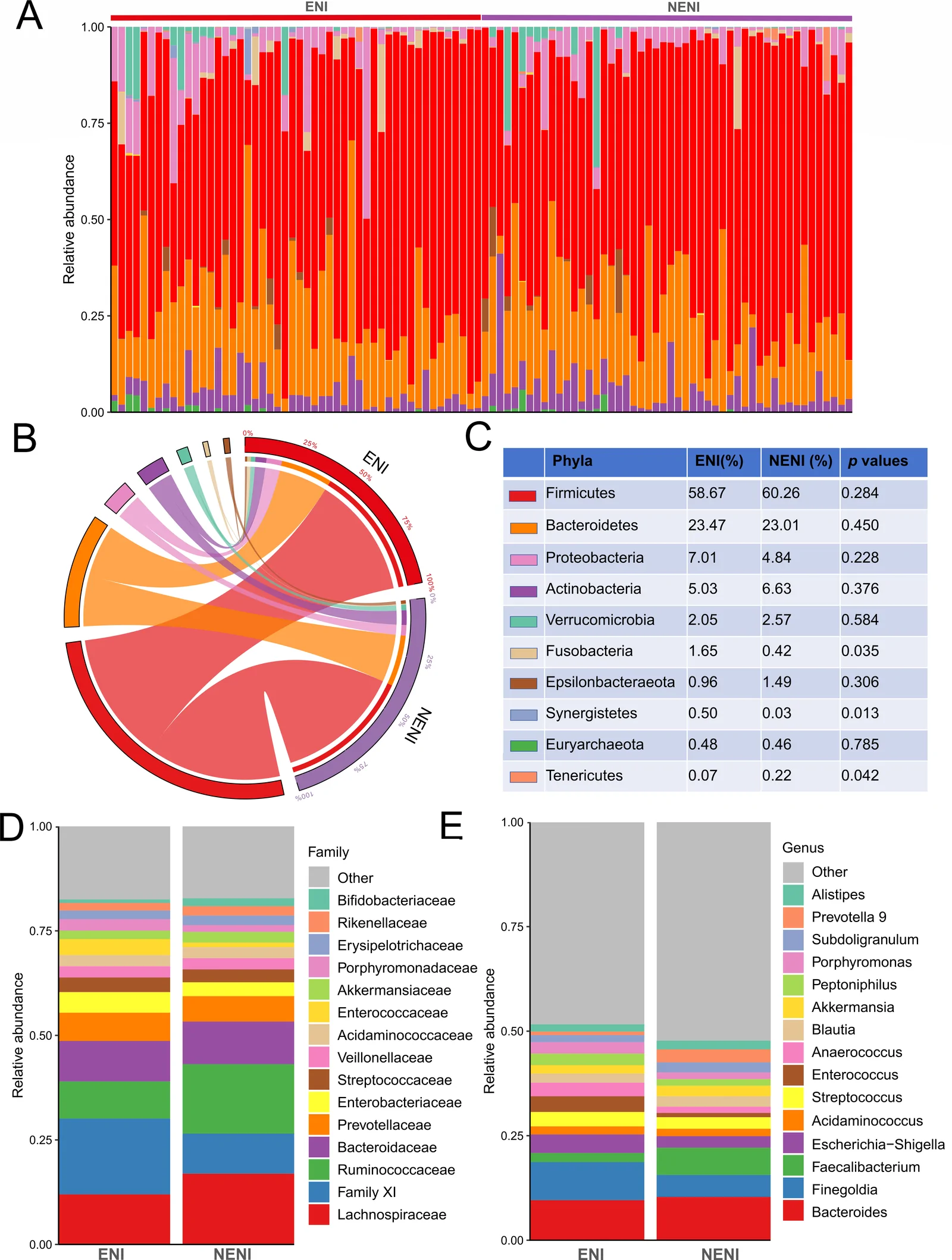
Microbial composition at different taxonomic levels. At the level of phyla, the composition of microbial communities was analyzed across all patients (**A**). Concurrently, the predominant bacteria are illustrated in a circos plot for ENI and NENI groups (**B**). The 10 leading microbial compositions at the phylum level differed between ENI and NENI groups (**C**). Furthermore, the distribution of microbial families (**D**) and genera (**E**) was investigated in both groups.

We generated 19,898,290 high-quality sequences from the samples, producing a median of 126,560 reads for each individual sample (99,486–139,580 reads). This extensive data collection allowed for the identification of 1,728 ASVs among the samples spanning 13 phyla, 70 families, and 264 genera of intestinal microorganisms (Fig. 3). To understand the differences in microbiota composition between individuals experiencing ENI and NENI, we then performed a comparison of relative taxonomic abundance assessment. At the phylum level, it was evident that the two groups shared a similar microbial structure composed primarily of five phyla: *Firmicutes*, *Bacteroidetes*, *Proteobacteria*, *Actinobacteria,* and *Verrucomicrobia* (Fig. 4A through C). When delving deeper into family-level composition, six families were identified as commonly dominant in the ENI and NENI groups: *Lachnospiraceae*, *Family XI*, *Ruminococcaceae*, *Bacteroidaceae*, *Prevotellaceae*, and *Enterobacteriaceae* (Fig. 4D). However, a greater distinction was observed at the genus level. In the NENI group, the top five genera, including *Bacteroides*, *Faecalibacterium*, *Finegoldia*, *Prevotella 9*, and *Streptococcus*, were noted. Meanwhile, the top five genera in the ENI group were *Bacteroides*, *Finegoldia*, *Escherichia-Shigella*, *Enterococcus,* and *Streptococcus* (Fig. 4E). For a comprehensive understanding of these variations, detailed relative abundance data can be found in Table S1. Additionally, heatmaps exhibiting the top 30 bacterial taxa at the family and genus levels were created and are presented in Fig. S1 and S2, providing a visual representation of the significant differences in microbial composition between the two groups.

### Reduced diversity of gut microbiota in patients with ENI

**Fig 5:**
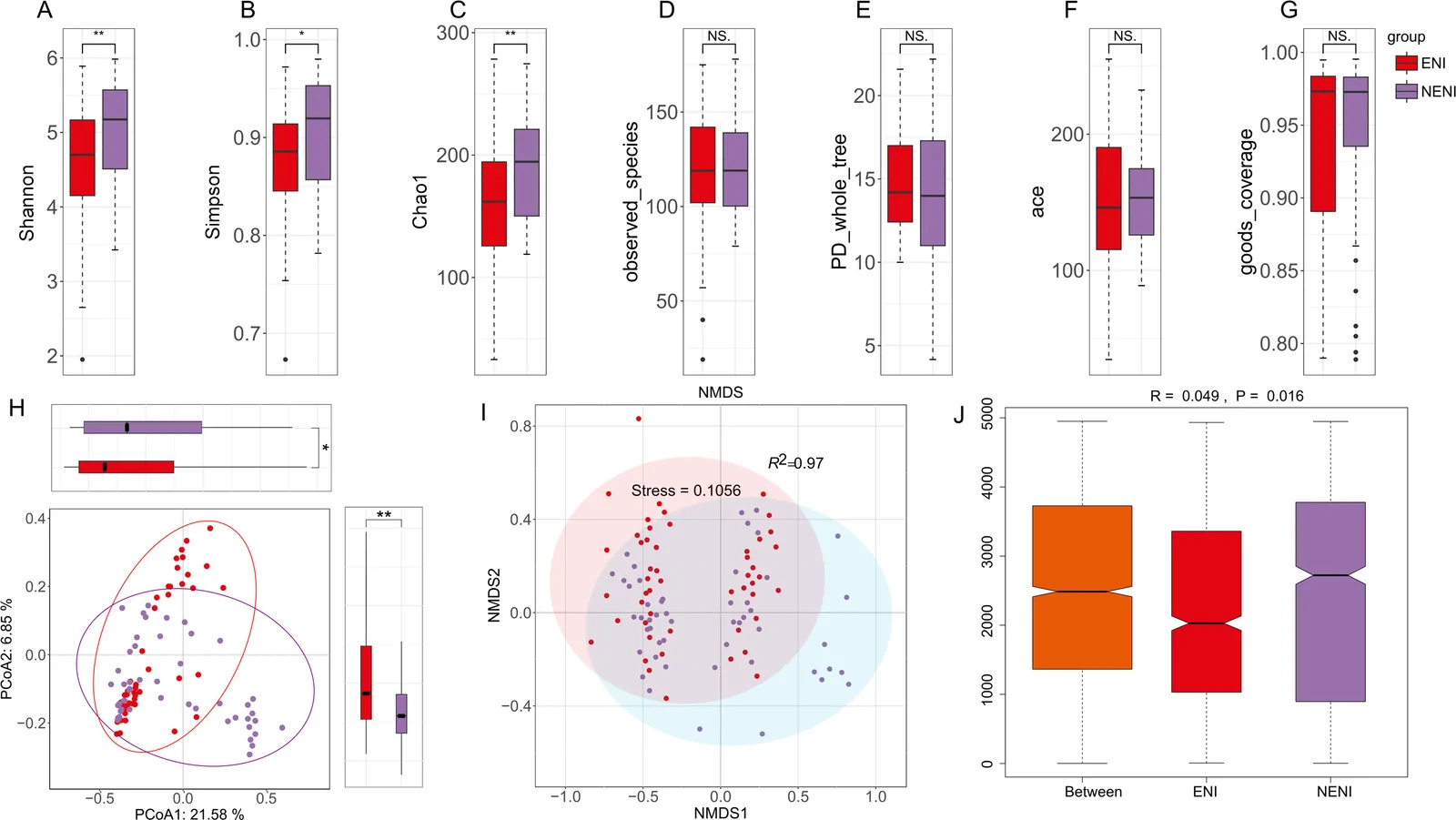
Intestinal microbial diversity between ENI and NENI groups. The comparison of various α-diversity indices is effectively illustrated through boxplots, and differences between groups were determined using an independent samples *t*-test for normally distributed data or the Mann-Whitney *U* test for non-normally distributed data (**A–G**). To visualize differences in microbial community structure, principal coordinate analysis (PCoA) was used, using unweighted UniFrac distances. The axes in the plot represent the first two principal coordinates, explaining 21.58% and 6.85% of the total variance, respectively. Each sample is represented by a dot, with different colors indicating different groups (**H**). Moreover, the variations in microbial composition are also illustrated by the NMDS based on Bray–Curtis distances. Samples with high gut microbiome compositional similarities are demonstrated as closely located points (**I**). The ANOSIM revealed significantly greater differences between groups than within groups (**J**). * 0.01 < *P* < 0.05; **P < 0.01.

ASVs were used for alpha diversity analysis to assess the diversity of intestinal microbiota. Various indices, including Shannon, Simpson, and Chao1, revealed a statistically significant decrease in microbial diversity in the ENI group compared to the NENI group, as illustrated in Fig. 5A through C. This finding highlighted a critical divergence in the microbial ecosystems between the two patient groups. However, non-significant differences were observed in other alpha diversity metrics, as displayed in Fig. 5D through G. Furthermore, beta diversity analysis offered additional insight into the structural variations within microbial communities. PCoA based on Bray–Curtis dissimilarities demonstrated a clear separation between the microbiota of the ENI and NENI groups. The first principal coordinate (PCoA1) accounted for 21.58% of the total variance, with statistical significance (*P* < 0.05), as revealed in Fig. 5H. Permutational multivariate analysis of variance (PERMANOVA) analysis indicated that group status (ENI vs NENI) explained 9.8% of the variance in microbiota composition (*R*² = 0.098, *P* = 0.012), providing a quantitative measure of effect size. This distinct separation underscored the unique microbial composition in each group. Complementing these results, NMDS analysis revealed significant compositional differences, supported by a low-stress value of 0.1056 < 0.2 (Fig. 5I), indicating a reliable data representation. Additionally, ANOSIM confirmed the robustness of these differences, with an *r*-value of 0.049 and a *P*-value of 0.016 (Fig. 5J). These findings suggest that the gut microbiota composition in patients with ENI is notably distinct and significantly altered compared to patients with NENI.

### Distinct gut microbial biomarkers in patients with ENI and NENI

**Fig 6:**
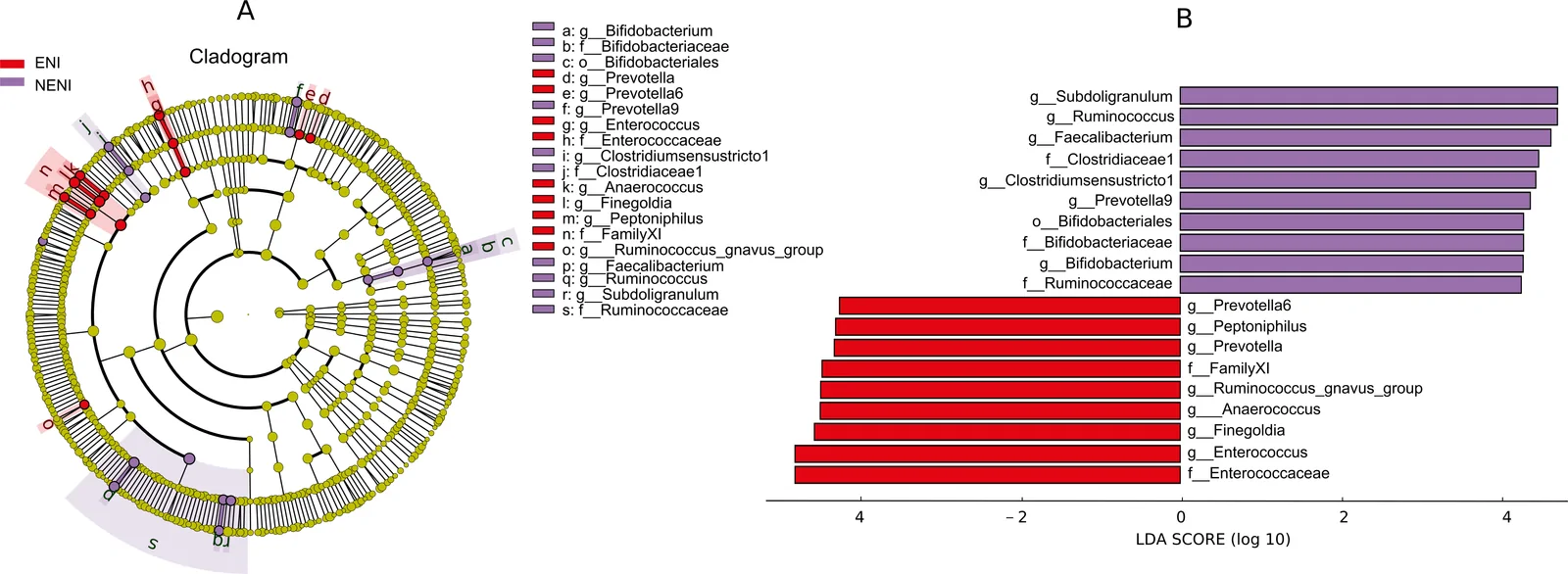
Cladogram (**A**) and LDA scores (**B**) illustrate the predominant microbial taxa found in patients diagnosed with ENI and NENI. Radiating from the innermost to outermost areas, several taxonomic levels are represented, spanning from phylum to genus. The nodes at each level signify the classifications of species. Yellow nodes indicate a lack of differentiation between groups, whereas nodes of varying colors indicate enrichment specific to their respective groups. Only taxa with an LDA score > 2 and *P* < 0.05, as determined by the Kruskal–Wallis test, were considered significantly discriminative and are shown.

Significant alterations in the microbial composition were observed between ENI and NENI groups. However, it became evident that relying solely on discriminant analysis was insufficient to identify the specific dominant taxa contributing to these differences. To address this limitation, a robust taxonomic comparison method, LEfSe, was used. Using this approach, 19 differentially represented microbial taxa were identified between the two groups, with 10 taxa enriched in the ENI group and 9 taxa enriched in the NENI group, as illustrated in Fig. 6A and B. The ENI group exhibited an increased abundance of genera potentially detrimental to host health, including *Enterococcus* and *Ruminococcus gnavus_group* (29, 30). Conversely, the NENI group had elevated levels of short-chain fatty acid (SCFA)-producing genera, including *Subdoligranulum* and *Faecalibacterium* (31, 32), which are commonly associated with gut health benefits.

### Identification of a microbial biomarker panel distinguishing ENI from NENI in emsICH patients

**Fig 7:**
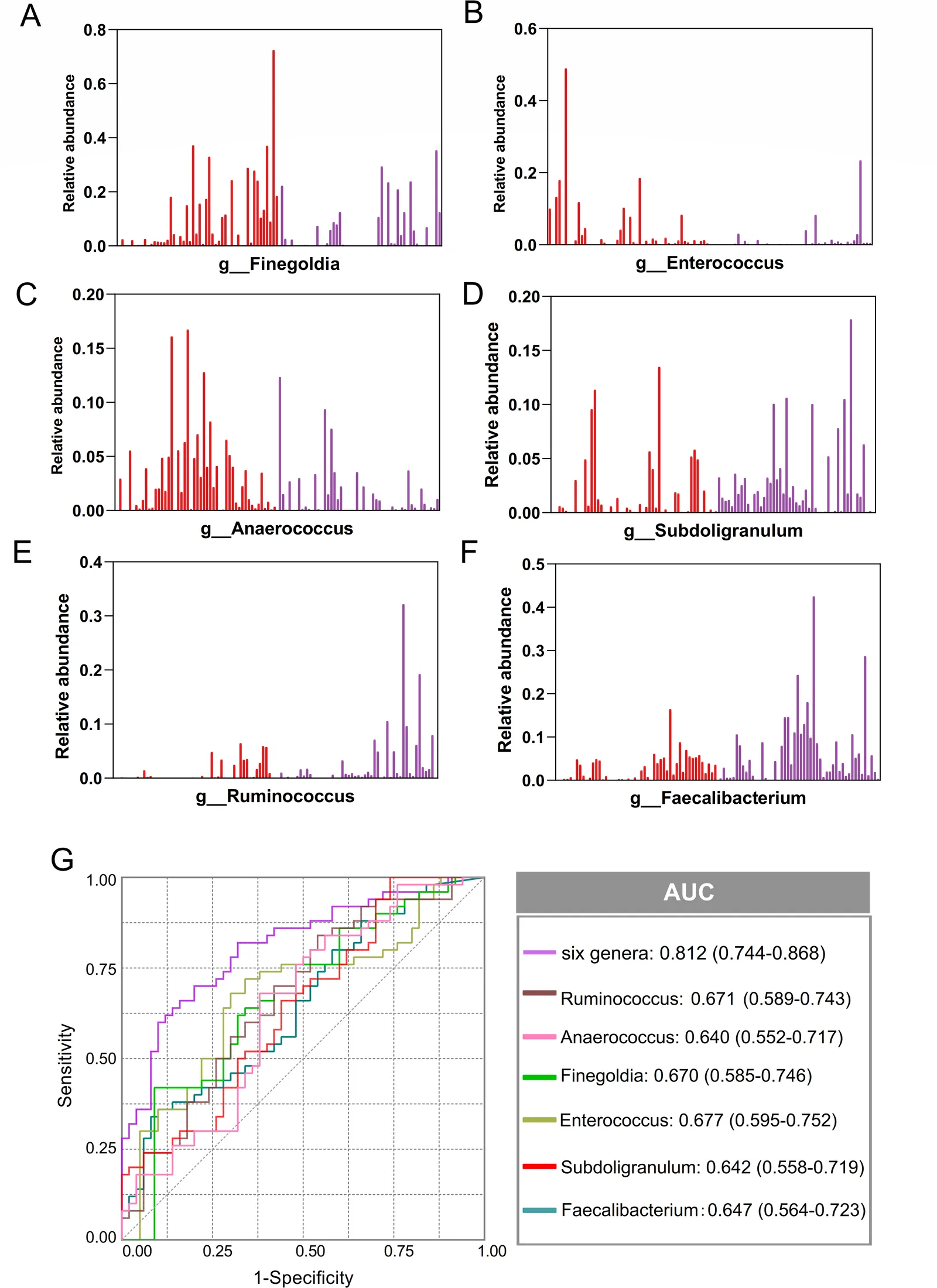
Microbial panel comprising six genera served as biomarkers for ENI (**A–F**). The abundance of these genera is presented in each sample (red for ENI and purple for NENI), including *Subdoligranulum, Ruminococcus_1, Faecalibacterium*, *Finegoldia*, *Enterococcus*, and *Anaerococcus*. ROC curves were generated to evaluate the ability of individual genera and their combinations to discriminate between ENI and NENI, with area under the curve (AUC) values and 95% confidence intervals shown (**G**).

A panel containing important genera associated with ENI and NENI was established for analysis. Six of the identified genera*—Subdoligranulum*, *Ruminococcus*, *Faecalibacterium*, *Finegoldia*, *Enterococcus*, and *Anaerococcus*—were the most common and abundant taxa identified among individuals in either patient group. This study revealed considerable variability in the abundance of these genera among different individuals, as demonstrated in Fig. 7A through F. To assess the predictive potential of these genera, ROC curve analysis was conducted, and the results revealed that each genus had good predictive ability (area under the curve [AUC]: 0.640–0.677). When all six genera were evaluated collectively as a unified biomarker panel, the AUC increased to 0.812, as presented in Fig. 7G. These results underscored the potential of this microbial signature as a reliable and non-invasive indicator for the prediction of ENI.

### Phenotypic alterations of gut microbiota in patients with ENI

**Fig 8:**
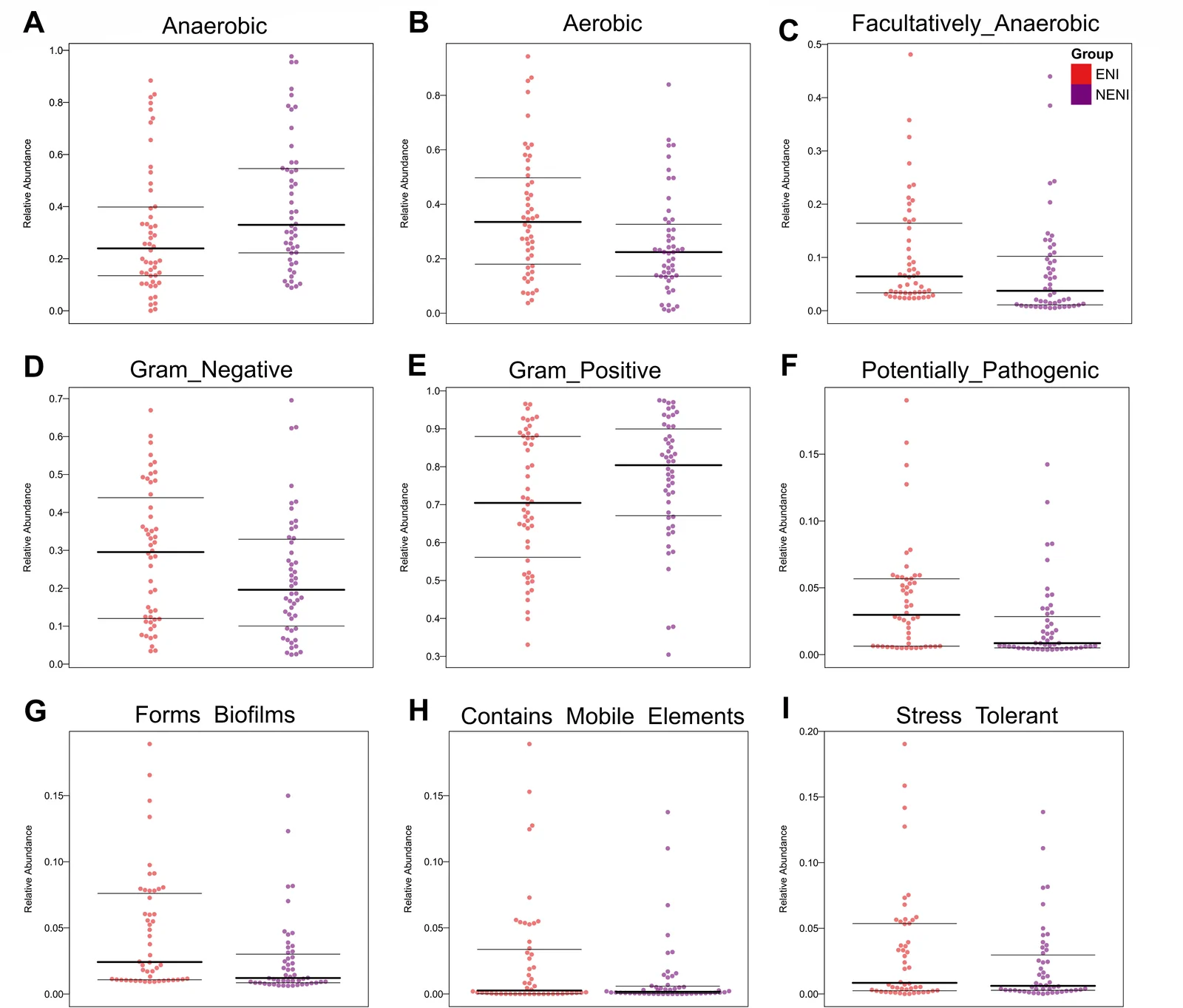
Phenotypic characteristics of gut microbiota in ENI and NENI. The BugBase tool was used to predict oxygen metabolism traits (**A–C**), structural and genetic traits (**D–F**), and functional and behavioral traits (**G–I**). Differences in predicted phenotypic features between groups were assessed using the Mann-Whitney *U* test, with *P* < 0.05 considered statistically significant.

To reveal the underlying associations between the gut microbiota and ENI, BugBase software was used to analyze the phenotypic traits of the microbes. Regarding oxygen metabolism traits, the abundance of the ENI group exhibited an increase in aerobic (*P* = 0.018) and facultative anaerobic (*P* = 0.009) bacteria and a decrease in anaerobic bacteria (*P* = 0.025). In terms of structural and genetic traits, the abundance of the ENI group revealed an increase in Gram-negative bacteria (*P* = 0.003), a decrease in Gram-positive bacteria (*P* = 0.003), and no difference in mobile elements (*P* = 0.14). Regarding functional and behavioral traits, the abundance of the ENI group exhibited an increase in biofilm formation (*P* = 0.039) and opportunistic pathogens (*P* = 0.008), with no difference in oxidative stress tolerance (*P* = 0.28) (Fig. 8A through I).

### Associations between gut microbiota and inflammatory indicators

**Fig 9:**
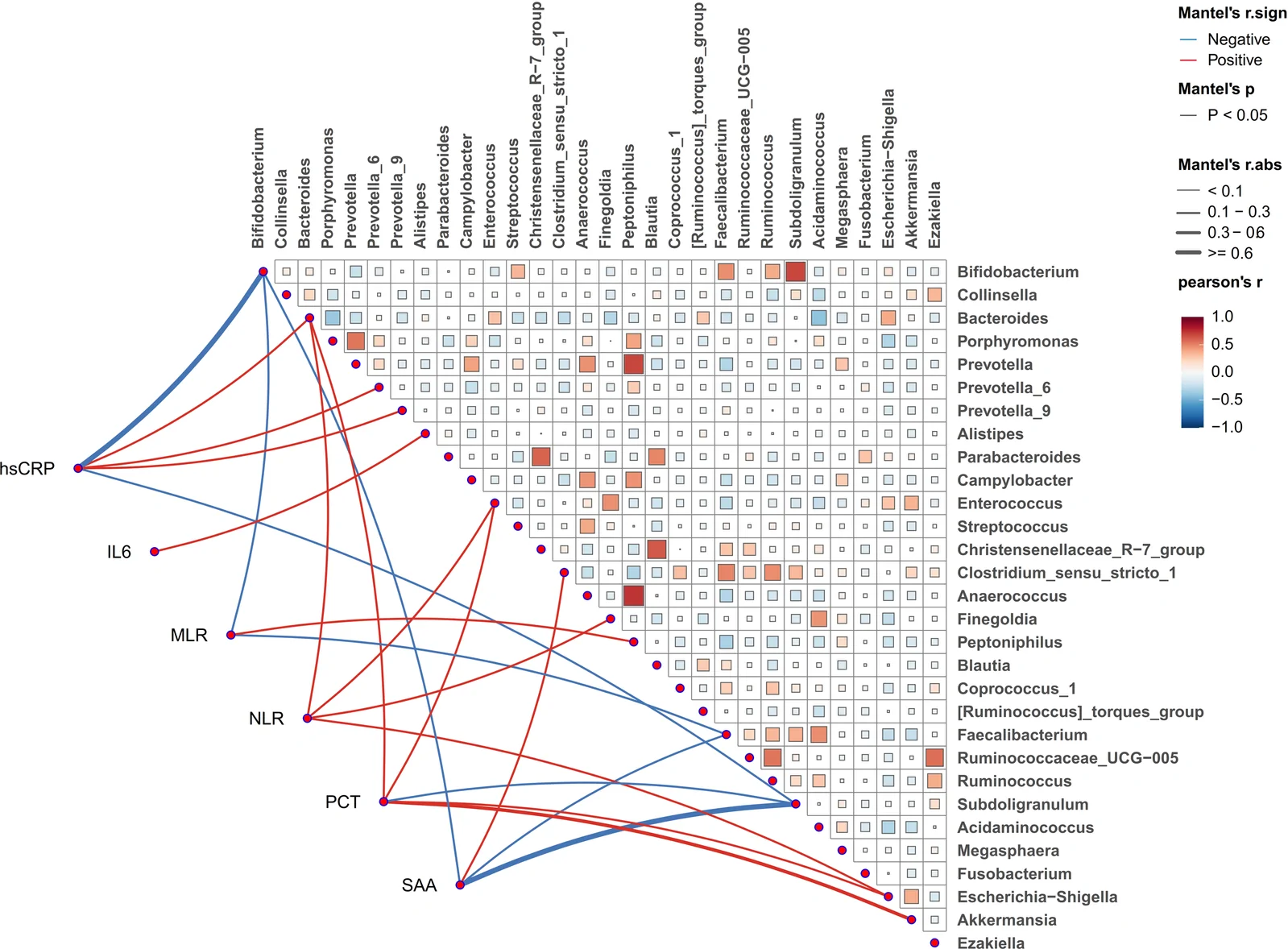
Analysis of correlation among diverse gut microbial genera and inflammatory indicators. The heatmap provides a visual representation of the interrelationships among the 30 genera that were abundant in ENI, based on Spearman’s rank correlation analysis. The intensity of the colors indicates the strength of the correlation among the different genera. In parallel, we used Spearman correlation analysis to explore the relationships between these genera and inflammatory indicators in patients with ENI. The red lines represent positive relationships, while the blue lines indicate the opposite. The thickness denotes the strength of the correlation. Statistically non-significant relationships are represented by gray dashed lines. Statistical significance was defined as an adjusted *P* < 0.05 after false discovery rate (FDR) correction.

To further investigate the relationship between gut microbiota alterations and inflammatory indicators of ENI, a genus-level correlation analysis was performed. Using Spearman correlation, associations between various gut taxa and systemic inflammatory indicators, including MLR, NLR, IL-6, SAA, PCT, and hs-CRP, were evaluated. The resulting correlation network is illustrated in Fig. 9. Notably, several taxa enriched in the NENI group, including *Bifidobacterium*, *Faecalibacterium*, and *Subdoligranulum*, were inversely correlated with inflammatory indicators, suggesting a potential protective role. Conversely, genera more abundant in patients with ENI, including *Finegoldia*, *Enterococcus*, and *Prevotella*, exhibited positive correlations with these inflammatory indicators. Meanwhile, PERMANOVA based on Bray–Curtis dissimilarity was performed to quantify the contribution of each inflammatory marker to overall gut microbiota composition. The analysis revealed that all six markers were significantly associated with variations in microbial community structure within the ENI cohort. Specifically, IL-6 explained 4.9% of the variance (*R*² = 0.049, *P* = 0.041), hs-CRP explained 8.8% (*R*² = 0.088, *P* = 0.003), MLR explained 5.1%, NLR explained 6.2%, SAA explained 4.6%, and PCT explained 7.5% of the variance (all *P* < 0.05) (Table S2). These results indicate that systemic inflammatory status is quantitatively associated with overall gut microbiota composition. Furthermore, strong inverse relationships were observed between health-promoting genera (*Subdoligranulum*, *Ruminococcus*, and *Faecalibacterium*) and inflammation-associated genera enriched in the ENI (including *Finegoldia*, *Enterococcus*, *Anaerococcus*, and *Prevotella*).

### Altered functional profile of gut microbiota in patients with ENI

**Fig 10:**
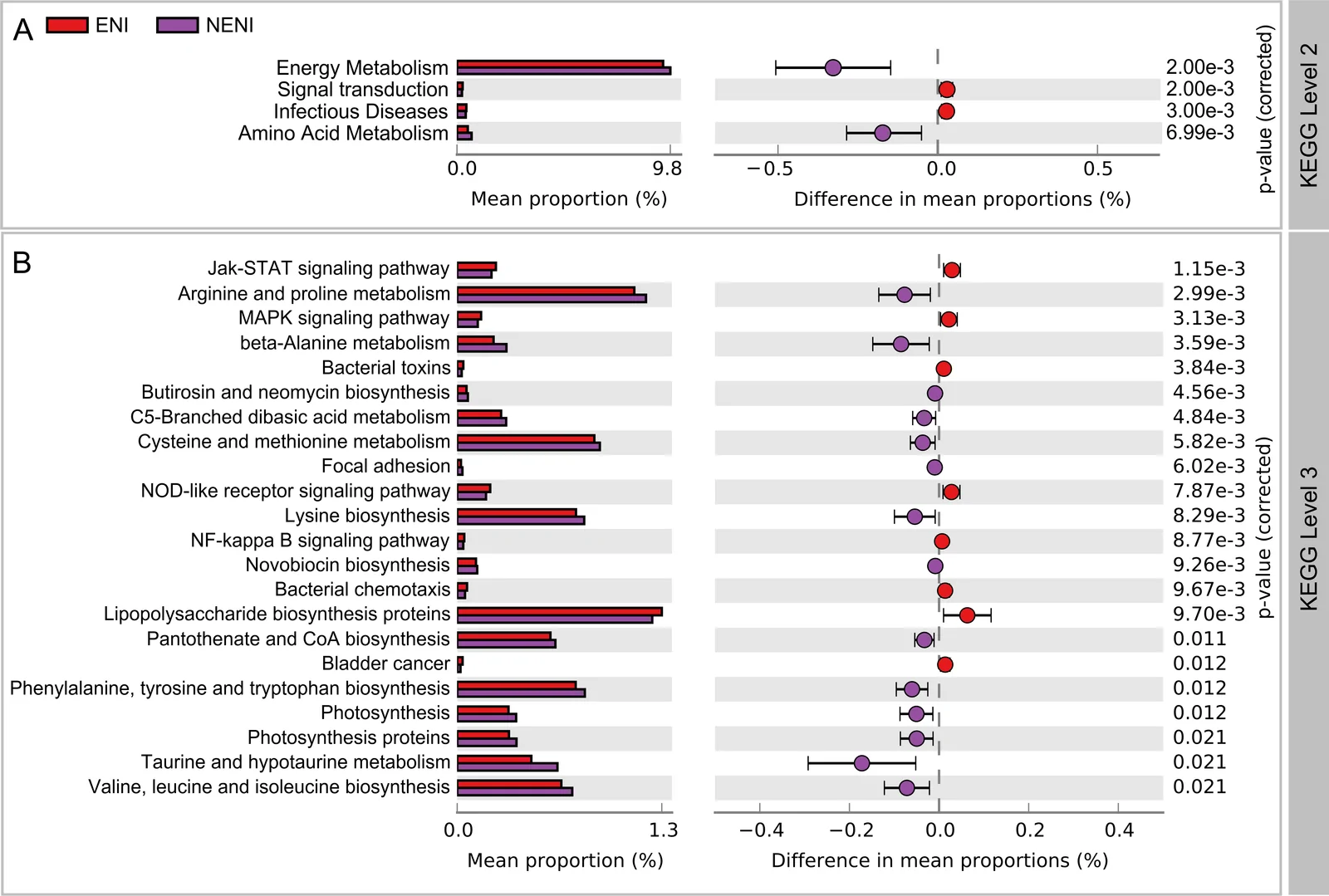
Predicted functional profiles of intestinal flora in ENI and NENI were conducted. This investigation revealed noteworthy changes in the pathways at KEGG level 2 (**A**) and the 20 pathways that were most significantly altered at KEGG level 3 (**B**), according to PICRUSt analysis. Group comparisons were performed using the Wilcoxon rank-sum test, and pathways with *P* < 0.05 were considered statistically significant.

To explore the functional shifts within the gut microbial communities, a predicted metagenomic analysis was performed using PICRUSt. This approach aligned individual ASVs with an integrated reference genome database to infer the functional potential of the microbial communities. Comparative analysis revealed the top 20 significantly different KEGG pathways between patients with ENI and NENI. As illustrated in Fig. 10A and B, patients with ENI exhibited a significant upregulation of microbial functions related to signal transduction, with notable enrichment in pathways implicated in inflammatory responses, including the Jak-STAT and MAPK signaling pathways. Conversely, key pathways involved in metabolism, particularly in energy metabolism, were markedly downregulated in the ENI group compared to the NENI group.

## DISCUSSION

Cerebral hemorrhage, among the most acute and critical neurological disorders, is a significant clinical challenge due to its high mortality and disability rates (33). Patients with cerebral hemorrhage frequently experience multiple system dysfunction, with intestinal dysfunction being particularly prominent (34–36). ENI is a common complication encountered during the nutritional support process for these patients, severely impairing nutrient absorption and utilization, which consequently delays recovery (3). Recently, the gut microbiota has garnered increasing attention for its role as an important “metabolic organ” and immune regulator in intestinal dysfunction following brain injury (37, 38). The bidirectional regulatory mechanism of the brain-gut axis suggests that cerebral hemorrhage can induce alterations in the gut microbiota. In contrast, gut microbiota can influence recovery from cerebral hemorrhage by modulating immune and metabolic responses (35, 39). This study systematically analyzes the changes in the structure and function of intestinal microbiota in patients with emsICH, alongside the detection of inflammatory factors, revealing a close association between microbiota dysbiosis and ENI. Furthermore, it explores the potential mechanisms and provides a theoretical basis for clinical nutritional therapy and microecological interventions.

This study demonstrates that the α-diversity of the intestinal microbiota in patients with ENI was significantly reduced, indicating impaired stability and richness of the microbial ecosystem (21). This decline leads to diminished microecological functions and an increase in potentially harmful bacteria. Specifically, the abundance of beneficial probiotics, including *Subdoligranulum, Ruminococcus1*, and *Faecalibacterium*, was markedly decreased. These bacteria have been reported to exert potential beneficial effects, primarily through butyrate production, based on previous studies (31, 40–42). Butyrate has also been shown in previous studies to serve as an energy substrate for intestinal epithelial cells and to be associated with enhanced expression of tight junction proteins, including occludin and TJP1, thereby preserving intestinal barrier integrity (43, 44). Concurrently, experimental evidence suggests that butyrate may promote the expansion of regulatory T cells via G protein-coupled receptors GPR43 and GPR109A, which inhibits inflammatory responses and mitigates the pro-inflammatory effects mediated by Th17 cells (45).

Additionally, *Ruminococcus* could regulate host immune responses and intestinal barrier function through the production of SCFAs, particularly propionate (45, 46). *Faecalibacterium*, a classic anti-inflammatory bacterium, could produce various anti-inflammatory metabolites (47), inhibit the nuclear factor kappa B (NF-κB) signaling pathway, and reduce the production of pro-inflammatory factors, including CRP, IL-6, and TNF-α (48, 49). Conversely, the abundance of harmful bacteria, including *Finegoldia*, *Enterococcus*, *Anaerococcus*, and *Ruminococcus gnavus*, significantly increases in the ENI group. *Finegoldia*, an opportunistic pathogen, could compromise the intestinal mucosal barrier by secreting lysozyme and proteases, which induce local inflammation and activate immune cells (50, 51). Moreover, *Anaerococcus* may alter the expression of genes that encode proteins associated with inflammatory response and contribute to eventual intestinal barrier dysfunction (52, 53). Moreover, *Enterococcus* could aggravate systemic inflammation by activating the host’s NLRP3 inflammasome and increasing the release of inflammatory cytokines, such as IL-1β (54–56). Meanwhile, *Ruminococcus gnavus* has been revealed to potentially promote intestinal inflammatory responses through 1,7-dimethyluric acid and histidine-related metabolites (30). In conclusion, the greater the increase in these types of pathogenic bacteria, the higher the endotoxin levels in the gut, which could aggravate systemic inflammatory responses (57), reduce gastrointestinal motility, and decrease the secretion of digestive enzymes (58). In other words, microbiota dysbiosis negatively affects the motor and secretory functions of the intestine, which may exacerbate ENI. Notably, the microbial patterns observed in the present emsICH cohort are consistent with findings reported in other ENI populations independent of intracerebral hemorrhage. Previous studies in critically ill patients have shown that ENI or feeding intolerance is associated with reduced microbial diversity, depletion of short-chain fatty acid–producing bacteria, and enrichment of potentially pathogenic taxa, together with impaired intestinal barrier function and altered inflammatory responses (14, 59). These shared features suggest that gut microbiota dysbiosis may represent a common pathophysiological component of ENI across different clinical contexts. Nevertheless, it should be acknowledged that the specific taxonomic shifts identified in this study may partly reflect the unique neuroinflammatory milieu and clinical characteristics of emsICH patients. In this study, by screening the core advantageous bacteria, we identified a biomarker panel with a high diagnostic value in predicting ENI risk in patients with emsICH. This finding could provide a novel approach for the early clinical identification of high-risk ENI patients. Specifically, stool samples can be collected at admission or preoperatively, and the abundance of the six identified genera can be quantified using high-throughput 16S rRNA sequencing or targeted qPCR. These microbial data, combined with systemic inflammatory markers and neurological function scores, can be incorporated into a predictive risk model to identify high-risk individuals, thereby guiding timely and individualized interventions, including nutritional management.

Although it is important to explore ENI-associated microbiota individually, assessing the microbial communities involved within an ecological network perspective is also important. In this study, we examined the dynamic interactions between different microorganisms and identified several significant, synergistic, and antagonistic interactions. These findings indicated that ENI may occur as a result of the co-regulation of all bacteria living within a complex and interconnected network in the gut rather than individual bacteria. More specifically, in the ENI group, we identified a number of Gram-negative bacteria (Fig. 8) that could secrete increased levels of LPS (60). LPS has been reported to potentially support the enrichment of facultative anaerobic pathogens (32). This process may damage the originally strictly anaerobic-dominated ecosystem and weaken the colonization of beneficial bacteria (32). This change may explain the observed increase in opportunistic pathogens, such as *Enterobacteriaceae*, and the decrease in SCFA-producing bacteria, such as *Subdoligranulum* and *Faecalibacterium* (31, 32). Meanwhile, the enrichment of opportunistic pathogens may exacerbate ENI through two primary pathways: (i) enhancing the capability of biofilm formation (61) and hindering the direct contact of nutrient substrates to the intestinal epithelium, leading to the accumulation of substrates and disturbed osmotic pressure, inducing ENI manifestations such as diarrhea and vomiting. (2) The enrichment of *Enterobacteriaceae* may induce neutrophil migration across the intestinal epithelial barrier, compromising intestinal integrity while simultaneously inhibiting the growth of SCFA-producing bacteria (32). A reduction in SCFA-producing bacteria leads to an increase in intestinal pH, further diminishing the inhibitory capacity against *Enterobacteriaceae*, thereby creating a vicious cycle of microbial dysbiosis (32). Moreover, in an effort to further understand the potential role of gut microbiota in ENI, we assessed the correlations between different microbial genera and clinical inflammatory indicators. We observed significant positive correlations between pro-inflammatory bacteria (such as *Finegoldia* and *Enterococcus*) and inflammatory indicators, while SCFA-producing bacteria revealed negative correlations. Overall, these findings suggest that gut microbiota is vital for modulating systemic inflammatory responses. Mechanistically, we hypothesized that pro-inflammatory bacteria, through endotoxin production, may activate host immune pathways, particularly the TLR4/NF-κB signaling pathway, leading to the release of inflammatory mediators, such as IL-6 and TNF-α (62). This pro-inflammatory milieu could suppress the growth of beneficial bacteria, particularly those producing SCFAs, exacerbating the microbial imbalance (63). This positive feedback loop may impair the intestinal barrier function and induce systemic inflammation, which may disrupt gastrointestinal motility and nutrient absorption, ultimately contributing to or worsening ENI. These dynamic changes highlight the complex mechanisms underlying intestinal microecological imbalances in patients with ENI and underscore the importance of restoring microbial homeostasis and regulating inflammatory responses in clinical settings. Future therapeutic strategies should aim to restore the gut microecology and control inflammation while addressing the patient’s nutritional needs to effectively improve ENI outcomes.

To further validate this hypothesis, we analyzed KEGG pathways. Surprisingly, the functional prediction of gut microbiota in patients with ENI suggested a relative enrichment of inflammatory signal transduction pathways, particularly in the Jak-STAT and MAPK signaling pathways. These are classical inflammatory pathways and have been revealed to be associated with various diseases, including stroke. For instance, Wang Li et al. (64) identified that Lcn2 gene knockout could influence the secretion of Gdf-1 in BV2 cells by modulating the JAK/STAT signaling pathway, thereby alleviating neuroinflammation and ultimately promoting recovery in patients with ICH. Fei et al. (65) discovered that the overexpression of the Homer1 gene could influence the phenotypic transformation of astrocyte A1 by inhibiting the activation of MAPK signaling, thus suppressing the inflammatory response in ICH. Conversely, pathways related to energy metabolism in ENI also exhibited a decrease in abundance, which may be attributed to the reduction of the microbial ecosystem in the ENI group. In summary, the complex interrelationship between ENI and the gut microbiota remains unclear. Disturbances in the microbiome-metabolism-inflammation junction may contribute to the onset and progression of ENI. It is reasonable to hypothesize that this condition may be induced by pro-inflammatory toxins or metabolites that disrupt the immune and inflammatory responses. Accordingly, microbial regulation could represent a significant adjunctive therapeutic strategy for preventing ENI in patients with emsICH. Numerous studies have demonstrated that supplementation with probiotics and prebiotics can restore gut microbiota diversity, promote SCFA production, strengthen the intestinal barrier, and mitigate endotoxin release and inflammatory responses (66–68). Consequently, we advocate the design of personalized enteral nutrition formulas that incorporate fiber, anti-inflammatory nutrients, and other functional components. By combining this approach with monitoring of gut microbiota biomarkers, dynamically adjusting nutritional plans may effectively reduce the incidence of ENI and enhance the recovery of patients with emsICH.

This study used a cross-sectional design, which poses challenges in fully elucidating the temporal dynamics and causal relationships associated with changes in gut microbiota. Accordingly, longitudinal follow-ups, together with clinical trials involving probiotics, prebiotics, or other microbiota-targeted interventions, should be conducted to evaluate the impact of modulating gut microbiota on ENI incidence in high-risk patients. Additionally, it should be noted that the functional implications discussed above are inferred from 16S sequencing, correlation analyses, and previously published literature, rather than from direct functional validation in the present study. Therefore, it is advisable to integrate multi-omics approaches, including metagenomics and metabolomics, to systematically analyze microbial metabolites and their interactions with host metabolism. The sample size in this study is relatively limited, necessitating an expansion of the sample pool and multi-center validation to enhance the generalizability and credibility of the findings. Future studies should investigate the associations between gut microbiota regulation in patients with emsICH, neural repair, and immune remodeling, thereby further elucidating the mechanisms of the gut-brain axis.

### Conclusion

This study revealed a strong association between ENI, inflammatory indicators, and gut microbiota dysbiosis in patients with emsICH. In patients with emsICH and ENI, the diversity of intestinal microbiota was reduced, accompanied by an increase in pro-inflammatory bacteria and a decrease in SCFA-producing bacteria, which may promote the occurrence of ENI by affecting immune-inflammatory responses and metabolic pathways. Moreover, the constructed microbiota biomarker panel has high value for the prediction of ENI, providing new strategies for early clinical identification and personalized treatment. In the future, microecological regulation combined with precise nutritional intervention is expected to improve ENI in patients with ICH and promote clinical recovery.

## Data Availability

The data presented in the study are deposited in the NCBI repository (https://www.ncbi.nlm.nih.gov/), accession number PRJNA1470415.

## SUPPLEMENTAL MATERIAL

Fig. S1 spectrum.03138-25-s0001.tif A heatmap illustrating the 30 most prominent taxa at the family level across all samples.

Fig. S2 spectrum.03138-25-s0002.tif A heatmap depicting the 30 leading taxa at the genus level from all samples.

Supplemental material spectrum.03138-25-s0003.docx Supplemental legends.

Table S1 spectrum.03138-25-s0004.docx The relative abundance of dominant gut microbiota between ENI and NENI groups.

Table S2 spectrum.03138-25-s0005.docx PERMANOVA analysis of associations between inflammatory markers and gut microbiota composition within the ENI cohort.

ASM does not own the copyrights to Supplemental Material that may be linked to, or accessed through, an article. The authors have granted ASM a non-exclusive, world-wide license to publish the Supplemental Material files. Please contact the corresponding author directly for reuse.
