# Supplementary figures and images for: Association of gut microbiota and inflammatory markers with enteral nutrition intolerance in patients with early-stage moderate-to-severe intracerebral hemorrhage
Source: Microbiol Spectr. 2026 May 29;14(7):e03138-25. doi: 10.1128/spectrum.03138-25 (PMC13340016; doi:10.1128/spectrum.03138-25)

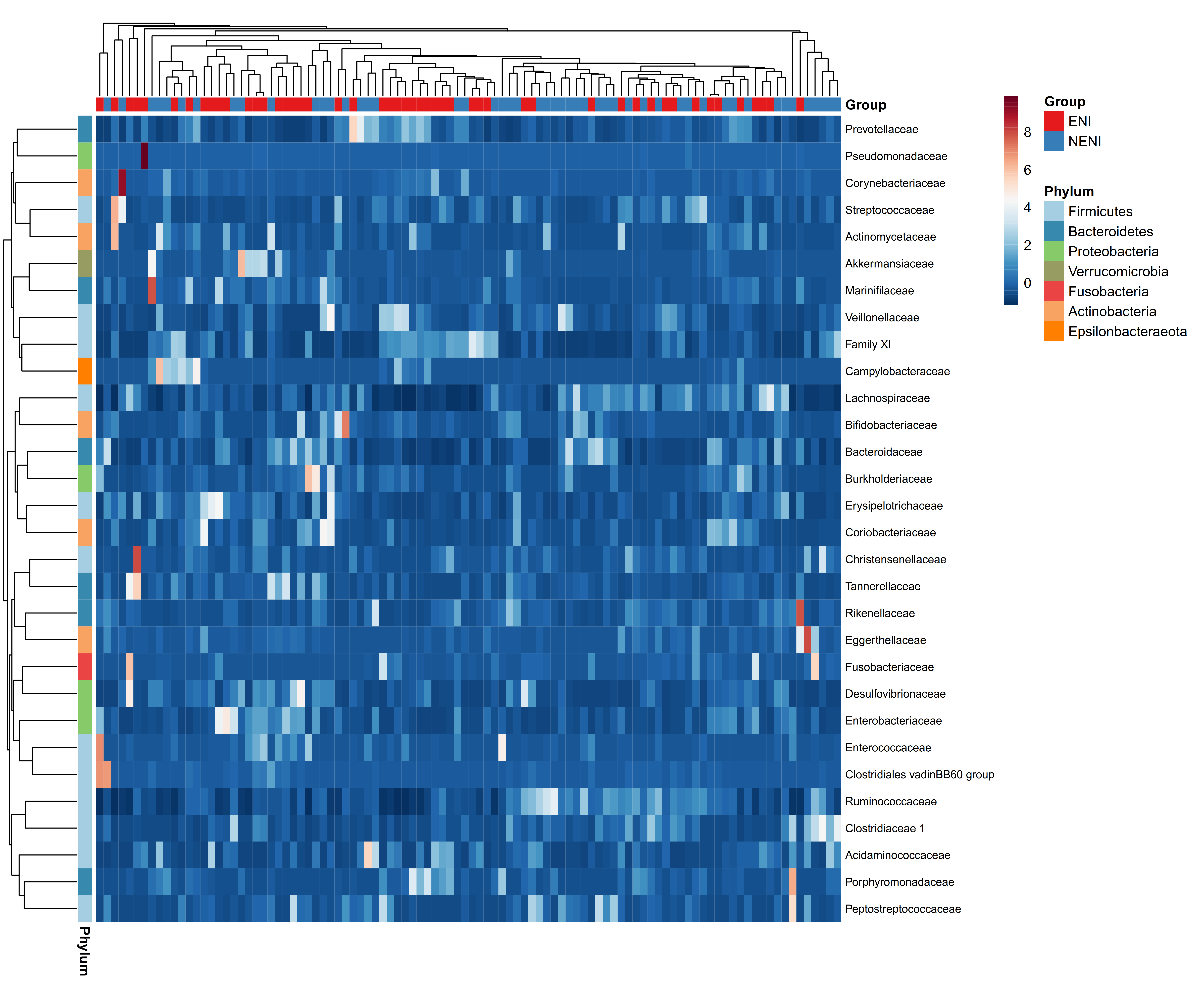

Supplement: Fig. S1 — A heatmap illustrating the 30 most prominent taxa at the family level across all samples. [file spectrum.03138-25-s0001.tif]

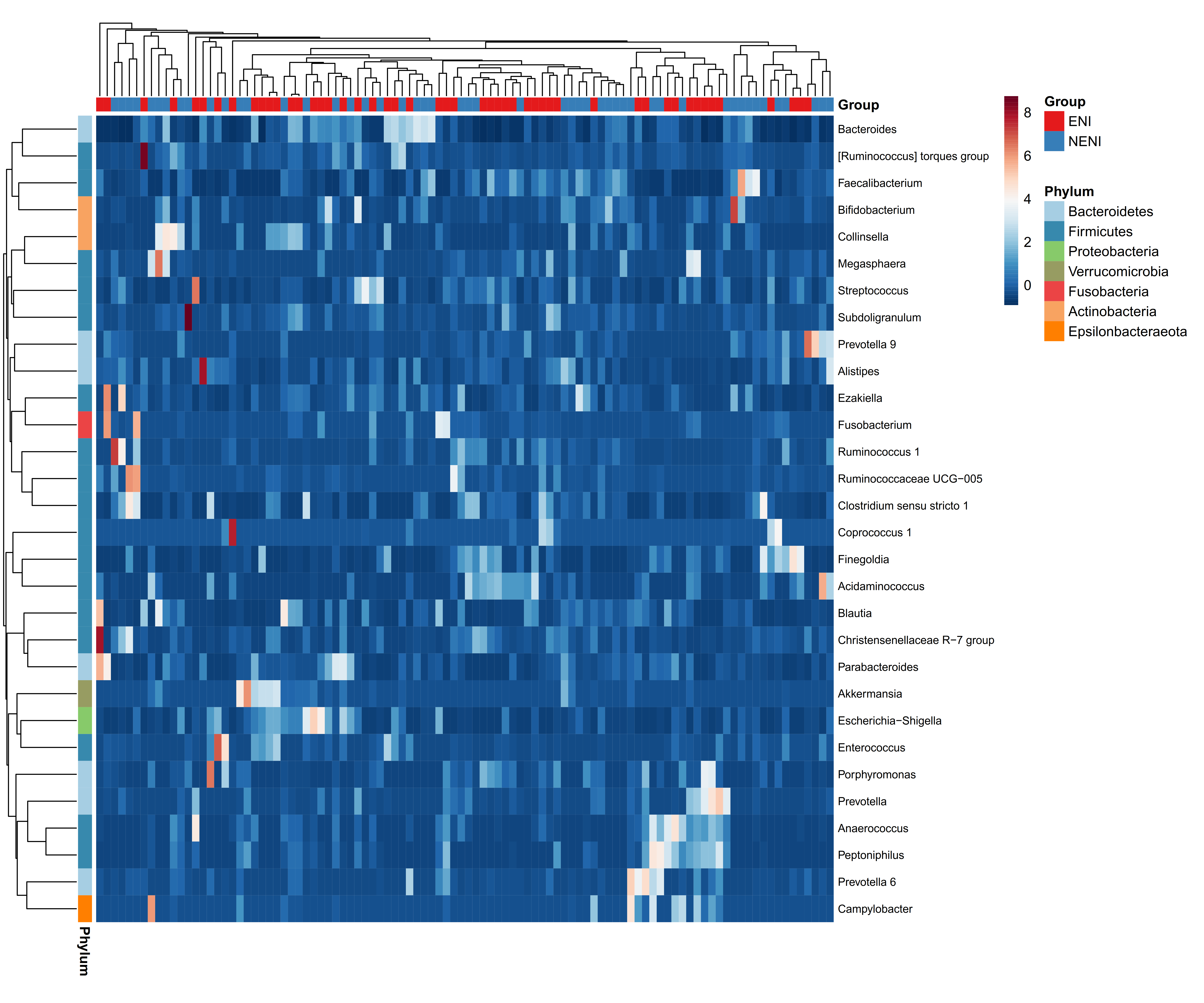

Supplement: Fig. S2 — A heatmap depicting the 30 leading taxa at the genus level from all samples. [file spectrum.03138-25-s0002.tif]
