# Supplementary material for: Association of gut microbiota and inflammatory markers with enteral nutrition intolerance in patients with early-stage moderate-to-severe intracerebral hemorrhage
Source: Microbiol Spectr. 2026 May 29;14(7):e03138-25. doi: 10.1128/spectrum.03138-25 (PMC13340016; doi:10.1128/spectrum.03138-25)
Supplement: Supplemental material — Supplemental legends. [file spectrum.03138-25-s0003.docx]

**Supplemental Legends**

**Supplementary Table S1.** Relative abundance of enriched gut microbiota between ENI and NENI.

**Supplementary Table S2.** Permutational multivariate analysis of variance (PERMANOVA) analysis of associations between inflammatory markers and gut microbiota composition within the ENI cohort.

**Supplementary Figure S1.** A heatmap illustrating the 30 most prominent taxa at the family level across all samples.

**Supplementary Figure S2.** A heatmap depicting the 30 leading taxa at the genus level from all samples.
