# Supplementary material for: Association of gut microbiota and inflammatory markers with enteral nutrition intolerance in patients with early-stage moderate-to-severe intracerebral hemorrhage
Source: Microbiol Spectr. 2026 May 29;14(7):e03138-25. doi: 10.1128/spectrum.03138-25 (PMC13340016; doi:10.1128/spectrum.03138-25)
Supplement: Table S1 — The relative abundance of dominant gut microbiota between ENI and NENI groups. [file spectrum.03138-25-s0004.docx]

**Supplementary Table S1. The relative abundance of** **[dominan](javascript:;)t gut microbiota between ENI and NENI groups.**

|  | ENI | NENI |
| --- | --- | --- |
| f_Family XI | 0.181205962 | 0.095700245 |
| f_Lachnospiraceae | 0.119779306 | 0.169321972 |
| f_Bacteroidaceae | 0.096614783 | 0.102059819 |
| f_Ruminococcaceae | 0.089060783 | 0.166172936 |
| f_Prevotellaceae | 0.067472704 | 0.060620879 |
| f_Enterobacteriaceae | 0.049484626 | 0.033196052 |
| f_Enterococcaceae | 0.037915062 | 0.010757558 |
| f_Streptococcaceae | 0.034883766 | 0.030921796 |
| f_Porphyromonadaceae | 0.027508304 | 0.016323636 |
| f_Acidaminococcaceae | 0.027021942 | 0.026421453 |
| f_Veillonellaceae | 0.026818008 | 0.026753474 |
| f_Erysipelotrichaceae | 0.020614971 | 0.022824705 |
| f_Akkermansiaceae | 0.020347164 | 0.025608174 |
| f_Rikenellaceae | 0.018305137 | 0.022526230 |
| f_Bifidobacteriaceae | 0.008298549 | 0.018865400 |
| g_Bacteroides | 0.096614783 | 0.102059819 |
| g_Finegoldia | 0.091197466 | 0.052748289 |
| g_Escherichia-Shigella | 0.044988472 | 0.027647607 |
| g_Enterococcus | 0.037915062 | 0.010757558 |
| g_Streptococcus | 0.034690606 | 0.028003282 |
| g_Anaerococcus | 0.033273846 | 0.015082429 |
| g_Prevotella_9 | 0.008375890 | 0.031644759 |
| g_Peptoniphilus | 0.028303644 | 0.016425565 |
| g_Porphyromonas | 0.027508304 | 0.016323636 |
| g_Blautia | 0.022155245 | 0.025056813 |
| g_Faecalibacterium | 0.021933227 | 0.065433459 |
| g_Akkermansia | 0.020347164 | 0.025608174 |
| g_Acidaminococcus | 0.0195791430 | 0.0177635392 |
| g_Alistipes | 0.017884187 | 0.021560274 |
| g_Subdoligranulum | 0.016799877 | 0.024935531 |

Abbreviations: ENI, enteral nutrition intolerance; NENI, non-enteral nutrition intolerance; p, phylum; f, family; g, genus.
