# Supplementary material for: Association of gut microbiota and inflammatory markers with enteral nutrition intolerance in patients with early-stage moderate-to-severe intracerebral hemorrhage
Source: Microbiol Spectr. 2026 May 29;14(7):e03138-25. doi: 10.1128/spectrum.03138-25 (PMC13340016; doi:10.1128/spectrum.03138-25)
Supplement: Table S2 — PERMANOVA analysis of associations between inflammatory markers and gut microbiota composition within the ENI cohort. [file spectrum.03138-25-s0005.docx]

**Supplementary Table S2. Permutational multivariate analysis of variance (PERMANOVA) analysis of associations between inflammatory markers and gut microbiota composition within the ENI cohort.**

| **Inflammatory marker** | **R^2^** value | **Variance explained (%)** | **p value** |
| --- | --- | --- | --- |
| hs-CRP | 0.088 | 8.8 | 0.003 |
| PCT | 0.075 | 7.5 | 0.006 |
| NLR | 0.062 | 6.2 | 0.012 |
| MLR | 0.051 | 5.1 | 0.021 |
| SAA | 0.046 | 4.6 | 0.028 |
| IL-6 | 0.049 | 4.9 | 0.041 |

Notes: PERMANOVA was conducted based on Bray–Curtis dissimilarity with 999 permutations.
